# The substitution effect of financial and non-financial incentives at different income levels in physician recruitment: evidence from medical students in China

**DOI:** 10.1186/s12909-024-05374-6

**Published:** 2024-05-09

**Authors:** Xinyan Li, Yue Zhang, Youli Han

**Affiliations:** 1 School of Public Health, Capital Medical University, No.10 Xitoutiao, Youanmenwai Street, Fengtai District, Beijing, 100069 China; 2 Qilu Hospital of Shandong University, No.107, Wen Hua Xi Road, Lixia District, Jinan, Shandong 250012 China

**Keywords:** Income level, Incentives, Substitution effect, Altruism, Discrete choice experiment, Medical student

## Abstract

**Background:**

Understanding how medical students respond to financial and non-financial incentives is crucial for recruiting health workers and attracting health talents in medical education. However, both incentives are integrated in working practice, and existing theoretical studies have suggested that various income levels may influence the substitution effect of both incentives, while the empirical evidence is lacking. Furthermore, little attention has been paid to the intrinsic motivation. This study aimed to explore the substitution effect of extrinsic incentives at different income levels, also taking intrinsic altruism into account.

**Methods:**

We used the behavioral data from *Zhang et al.*’s experiments, which involved discrete choice experiments (DCEs) to assess the job preferences of medical students from six teaching hospitals in Beijing, China. The incentive factors included monthly income, work location, work environment, training and career development opportunities, work load, and professional recognition. Additionally, a lab-like experiment in the medical decision-making context was conducted to quantify altruism based on utility function. Furthermore, we separated the choice sets based on the actual income and distinguished the medical students on altruism. The willingness to pay (WTP) was used to estimate the substitution effect of incentives through conditional logit model.

**Results:**

There was a significant substitution effect between non-financial and financial incentives. As income increased, non-financial incentives such as an excellent work environment, and sufficient career development became relatively more important. The impact of the increase in income on the substitution effect was more pronounced among individuals with higher altruism. Concerning the non-financial incentive work environment, in contrast to the growth of 546 CNY (84 USD) observed in the low-altruism group, the high-altruism group experienced a growth of 1040 CNY (160 USD) in the substitution effect.

**Conclusions:**

The increase in the income level exerted an influence on the substitution effect of non-financial incentives and financial incentives, especially in high-altruism medical students. Policymakers should attach importance to a favorable environment and promising career prospects on the basis of ensuring a higher income level. Medical school administrations should focus on promoting altruistic values in medical education, enhancing talent incentives and teaching strategies to encourage medical students to devote themselves to the medical professions.

**Supplementary Information:**

The online version contains supplementary material available at 10.1186/s12909-024-05374-6.

## Introduction

The health workforce as a primary resource to meet population healthcare needs, has become a central focus in healthcare reform. A growing body of evidence suggested that the quality of health services depended on highly motivated health workforce members who were satisfied with their jobs, and therefore stayed at their positions [1, 2]. In recent years, many countries have been faced with insufficient health workers and poor health indicators to achieve population health goals, especially in low and middle-income countries. (WHO, 2006). One effective approach to address this challenge is to implement incentive schemes [3]. Medical students, regarded as the future workforce of medical professions, have been urged to receive meaningful incentives aimed at fostering motivation to study medicine and dedicate themselves to healthcare careers [4]. Understanding how medical students respond to incentives is crucial for enhancing the health worker recruitment, and developing talent incentive strategies for guiding professionals in medical education [5].

In the guideline for incentives to health workers, WHO categorizes extrinsic incentives into two major groups, financial and non-financial ones [6]. Financial incentives, typically integral to the employment contract, such as salary, allowances, and bonuses are designed to fulfill the needs of employees [7]. The success of financial incentives in attracting medical professionals has been long discussed [8, 9]. In contrast to the commonly held belief, Ellis and Pennington (2004) suggested that financial incentives were observed to exert only a short-term effect on the motivation level [10]. Solely relying on financial incentives are insufficient as a motivator for medical students’ job choice, for sustainability, schemes must be completed by non-financial incentives [11]. Hence, instead of a range of financial incentives, non-financial elements seem to be necessary.

Non-financial incentives, defined as incentives that transfer monetary values or equivalents, generally include health workers’ job promotion, recognition, training and development, and other managerial factors [12, 13]. Moreover, a range of evidence indicated that there was a substitution relationship between financial and non-financial incentives [14]. Both financial and non-financial incentives likely contribute to the motivation of medical profession, and it now appears to be sufficient. Nevertheless, non-financial incentives are occasionally integrated with financial incentives. It is difficult to attribute outcomes to single incentives only, as the interactive effects may be quite complicated. What crucial is understanding the relative importance of incentives.

A concern emerges from recent needs-theories, indicating that while the fundamental human needs served as motivators, extrinsic motivators possessed a limited utility and had the potential to diminish individual effectiveness [15]. Additionally, from the economic perspective, there exists a threshold where additional financial incentives will have a limited effect [16]. The emerging empirical studies have found that the relationship between income and job satisfaction has become to be asymptotic when modeled as a curvilinear function [17, 18]. Consequently, there is a necessity to investigate the substitution effect between financial and non-financial incentives at various income levels.

Many studies have based on the DCEs to identify the substitution effect of different incentive factors on medical students’ job choices [19, 20]. Recent DCEs have gradually brought to attention that the financial rewards may not be as fundamental as previously believed [21]. Simultaneously, theories of organizational behaviors have emphasized the significance of intrinsic motives. Altruism, as an important intrinsic motivation in the principal-agent relationship between physicians and patients [22], also holds a pivotal role in physicians’ responses to incentives [23]. Zhang et al. (2023) investigated the effect of intrinsic altruism on the influence of extrinsic incentives on the job preference of medical students [24]. However, the substitution effect of external incentives has not received adequate attention regarding the impact of income levels. Furthermore, despite the significance of intrinsic motivation, little is known about whether altruism can be attributed to the fluctuation of substitution effect across different income levels.

This study contributes to filling the gap in the evidence on the link between the substitution effect of extrinsic incentives in medical students’ job choices across different income levels, combining with the intrinsic motivations. Firstly, we estimate the substitution effect of external factors relying on the data in Zhang et al.’s [24] DCEs among medical students, specifically financial and non-financial incentives, taking into account the actual design of basic income levels. Additionally, altruism measured by a lab-like economic experiment is included to examine whether it contributes to the income level and the substitution effect.

Our structural estimation provides two main results. Firstly, we find that at higher income level, medical students place a greater emphasis on non-financial incentives, which results in an elevated substitution effect between non-financial and financial incentives. Secondly, altruism has the potential to impact the extent of substitution effect at different income levels, with a greater variation observed in individuals with higher altruism. These findings offer principles regarding appropriate incentives for physicians’ recruitment, and developing talent incentives and teaching strategies to encourage medical students to dedicate themselves to health careers in medical education.

## Methods

### Research hypotheses

The process of medical job choice can be served as a manifestation of the substitution effect between financial and non-financial incentives, and the marginal rate of substitution (MRS) between different incentives, provides insights into the trade-offs in job preferences. Our framework operated under the assumption that medical students would opt for a working condition with the highest utility considering both financial (*M*) and non-financial (*N*) incentives at different income levels ($i$).

Additionally, an increase in the income may result in diminishing marginal utility, ultimately leading to the ineffectiveness of financial incentives [25]. The relationship between income level and the utility function for financial incentives would exhibit a curve, reaching its maximum at $$ {i}^{*}$$. Furthermore, as basic needs satisfaction increased, the value attributed to non-financial incentives also increased [26] (formula in Appendix Table S1). Thus, we formulated the following hypothesis:

#### Hypothesis 1

As income reaches a certain point, the substitution effect between non-financial and financial incentives is higher than that at the lower income level.

In the principal-agent relationship between physicians and patients, altruism represented the extent to which emphasis was placed on patient benefit under the assumption of utility maximization [27]. Individuals with higher altruism exhibited a higher weight attachment to patient benefits over their own financial profits. Furthermore, medical students with higher altruism paid more attention to non-financial incentives [24] (formula in Appendix Table S2). Thus, we formulated the following hypothesis:

#### Hypothesis 2

Medical students with higher altruism demonstrate a more pronounced degree of variation in the substitution effect of non-financial and financial incentives across different income levels.

**Fig. 1:**
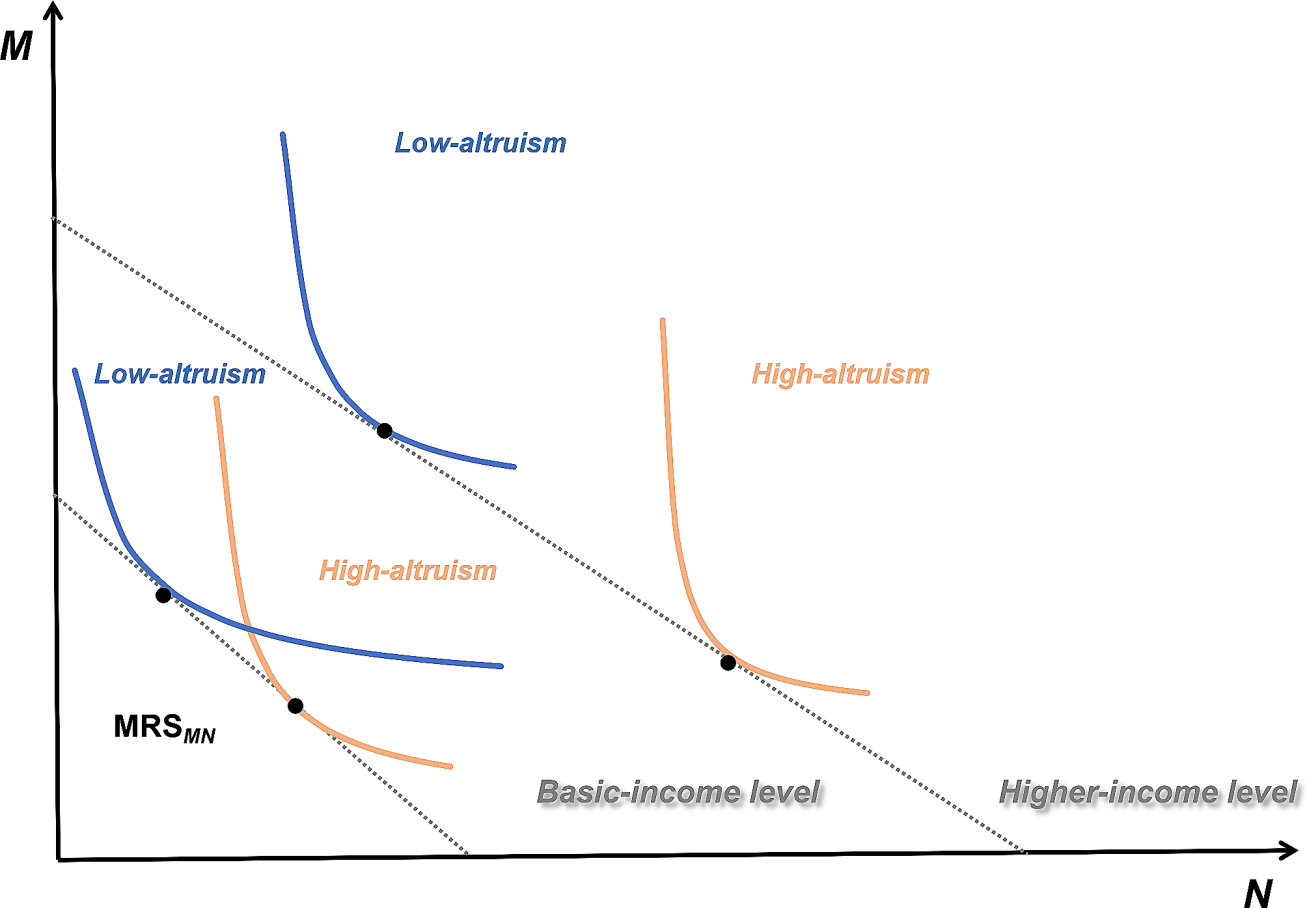
The theoretical hypothesis derivation schematic. The blue curve: utility function of non-financial (*N*) and financial (*M*) incentives in low-altruism group across different income levels; The yellow curve: utility function of non-financial (*N*) and financial (*M*) incentives in high-altruism group across different income levels; The tangent point on the curve: The substitution effect of non-financial and financial incentives ($$ {MRS}_{MN}$$)

The schematic diagram depicting the derivation of theoretical hypotheses is presented in Fig. 1.

### Behavioural data

Our experimental data stem from the DCEs combined with a lab-like experiment of Zhang et al. (2023) [24] who analyzed medical students with different levels of altruism regarding extrinsic job attributes. In total, 741 medical students were integrated into the formal analysis, which were selected through cluster sampling from six teaching hospitals in Beijing, China. Participants were required to complete a self-reported questionnaire consisting of basic personal information, experiment on altruism, and DCEs job choice tasks.

DCEs were conducted to estimate the substitution of extrinsic incentive factors, particularly focused on the interplay between financial incentives and non-financial incentives. The final six critical factors related to the recruitment of medical students were as follows: monthly income, work location, work environment, training and career development opportunities, workload, and professional recognition. The details of incentives and their corresponding levels are presented in the Appendix Table S3-S4.

The economic experiment has been demonstrated to facilitate the measurement of medical students’ altruism [24, 28]. Specifically, a laboratory-like experiment was designed in the context of medical decision-making, all medical students took the role of physicians and determined the quantity (*q*) of medical services, thereby influencing their self-profit (*π*(*q*)) and patient benefit (*B*(*q*)). Each subject, in accordance with the present parameters, selected quantities form the set *q*∈[0, 1, 2, 3, 4……10] for nine different patients, categorized into three types of illness *k*∈[*A*, *B*, *C*] and three levels of severities *l*∈[*x*, *y*, *z*]. Detailed experimental screen and parameter descriptions are available in Appendix Fig. S1 and Table S5. After the experiment, the physician profits were paid to the medical students and patient benefits were donated to the Red Cross Society of China to aid real patients. Based on this behavioral data, medical students’ altruism *α* can be quantified by evaluating the extent to which utility-maximizing physicians attach importance to *B*(*q*) in the trade-off between *π*(*q*) and *B*(*q*). Specifically, altruism *α*$\in$[0, 1] was calculated by using the first-order condition of utility function of physicians: *U*(*q*) = (1 − *α*) *π*(*q*) + *α B*(*q*), whereby a larger α indicated higher altruism. *α* = 0 represented physician’s pursuit of profit-maximization, and *α* = 1 represented physician’s full consideration of patient benefit. More details on experiment can be found in Zhang et al. (2023) [24] and Appendix Table S5-S6.

### Data analysis

Random Utility Models (RUMs) have been extensively utilized for the analysis of DCEs. Following the principle of the Akaike information criterion (AIC) and Bayesian information criterion (BIC) [29], we applied a conditional logit model for regression analysis (refer to the Appendix Table S7 for the estimation results of the mixed logit model). The monthly salary, representing the financial incentive was specified as a continuous variable, while all non-financial incentives were set as categorical variables. Based on the estimated coefficients, we calculated the substitution of extrinsic incentives by WTP, indicating the amount of money participants were willing to receive in exchange for a corresponding enhancement in a particular non-financial incentive.

The monthly income level for financial incentives was established based on the 2020 Hospital Salary Research Report in China. The average annual income of junior title doctors in the sample hospitals was approximately 110,000 CNY^1^ (16,923 USD), equivalent to income of about 9000 CNY (1384 USD) per month. Adjustments, both upward and downward, were made by 3000 CNY (461 USD) from this base. Since the study aimed to analyze based on income levels, we assumed that the average level of monthly income 9000 CNY (1384 USD) as $$ {i}^{*}$$, further distinguished between the choice set of basic-income level (6000 CNY vs. 9000 CNY) and higher-income level (9000 CNY vs. 12,000 CNY) to calculate the WTP, thereby confirming the variations in substitution effect.

The equation for the substitution of non-financial incentive $$ x$$ and financial incentive income was formulated as follows.

$$ WTP\left(x\right)=-\frac{\partial U/\partial x}{\partial U/\partial financial-incentive}=-\frac{{\beta }_{x}}{{\beta }_{financial-incentive}}$$

## Results

### General characteristics

A total of 741 medical students successfully completed the foundational medical knowledge learning and advanced to the clinical internship. The participants had an average age of 24 years old, with 422 females (59.7%), and 511 (68.9%) being postgraduate students. The demographic details are presented in Table 1.

**Table 1:** General characteristics for medical students (*N* = 741)

Demographics	Analysis Sample (%)
**Age**	24.0 (± 2.7)
**Gender**	
Women	442 (59.7)
Men	299 (40.3)
**Level of education**	
undergraduate	230 (31.0)
postgraduate	511 (68.9)
**Birthplace**	
Township or village	189 (25.5)
County	120 (16.2)
City	432 (58.3)
**Single child**	
No	293 (39.5)
Yes	448 (60.5)
**Strategic career planning**	
Engage in health-related work	313 (42.1)
Engage in non-health related work	5 (0.7)
Continue education	416 (56.1)
Others	7 (0.9)

### Estimations of the job preferences

In estimation of all analyzed samples (Model 1 in Table 2), we observed that the coefficients of all extrinsic incentives were statistically significant positive, indicating that all incentives had an effect on job preference. Concerning non-financial incentives, the participants manifested the greatest job preference for a job with a better location ($\beta$=0.820, *P <* 0.001), followed by a favorable work environment ($\beta$=0.554, *P <* 0.001), and ample training and career development ($\beta$=0.529, *P <* 0.001). The workload and professional recognition were relatively less important.

**Table 2:** Estimation for job preferences

	(1) Analysis Sample		(2) 6000 vs. 9000 CNY		(3) 9000 vs.12,000 CNY	
Incentive factors	Coeff.	SE	Coeff.	SE	Coeff.	SE
Monthly income	0.000379***	8.80e-06	0.000449***	3.26e-05	0.000373***	3.04e-05
Work location: village or township (ref)	0.820***	0.0251	0.737***	0.0730	0.834***	0.0495
Work environment: poor (ref)	0.554***	0.0237	0.497***	0.0539	0.783***	0.0705
Training and career development opportunities: insufficient (ref)	0.529***	0.0258	0.489***	0.0662	0.686***	0.0510
Workload: 60 h/week (ref)	0.273***	0.0223	0.238**	0.0749	0.352***	0.0565
Professional recognition: low (ref)	0.393***	0.0236	0.205**	0.0627	0.439***	0.0554
N	741		741		741	
Observation	26,559		8,796		8,850	
Log likelihood	-7333.7445		-2573.8478		-2373.907	
Pseudo R^2^	0.246		0.2009		0.2675	
LR χ^2^	4784.54		1294.57		1734.0	
Prob > χ^2^	< 0.0001		< 0.0001		< 0.0001	
AIC	14681.49		5161.696		4761.814	
BIC	14738.8		5211.27		4811.431	

^***^P < 0.001, ^**^P < 0.01, ^*^P < 0.1. Coeff: mean estimated coefficient; SE: standard error; AIC: Akaike Information Criterion; BIC: Bayesian Information Criterion. Since monthly income was treated as a continuous variable, its estimated coefficient was less than 0.001. The exchange rate for USD/CNY = 6.5.

To investigate the influence of various income levels on the substitution effect, we made distinctions among specific choice set of income. At the basic-income level (Model 2 in Table 2), the coefficient of monthly income ($\beta$=0.000449, *P <* 0.001) surpassed that observed in the overall analysis sample ($\beta$=0.000379, *P <* 0.001), whereas the coefficients of other non-financial incentives were comparatively lower than those in overall sample. Conversely, the opposite situation was observed at the higher-income level (Model 3 in Table 2).

### The substitution effect of non-financial and financial incentives

Based on the above estimation and the calculation of WTP, we estimated the substitution between financial and non-financial incentives. The results showed a significant difference in the substitution effect between two income levels, with a greater WTP observed at the higher-income level compared to the basic-income level.

**Fig. 2:**
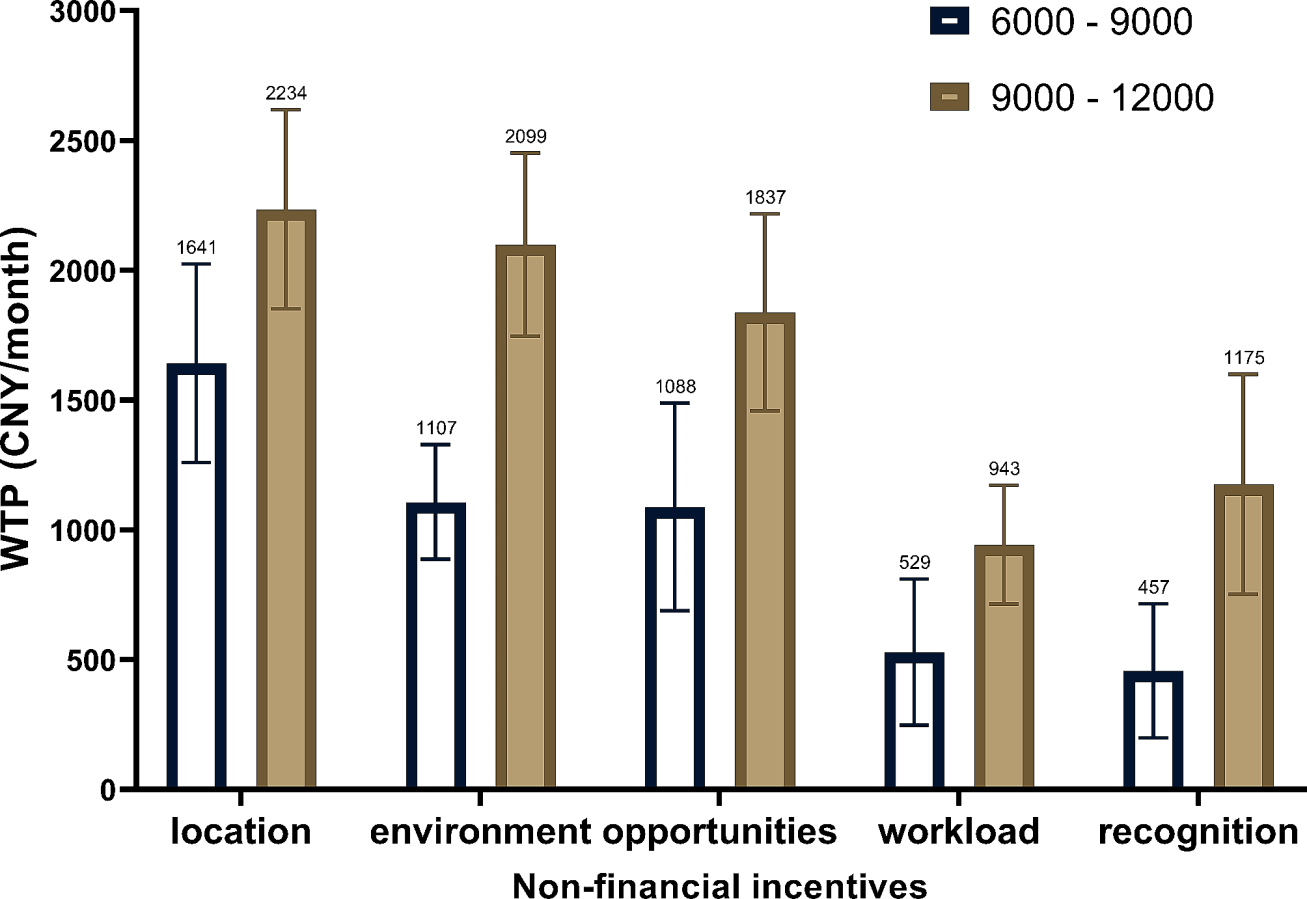
The substitution of non-financial and financial incentives. location: Work location; environment: Work environment; opportunities: Training and career development opportunities; workload: Workload; recognition: Professional recognition. The exchange rate for USD/CNY = 6.5

In the basic-income level, individuals were only willing to allocate 1641 CNY (252 USD) per month for workplace enhancement. However, as their monthly income elevated, the substitution effect increased to 2234 CNY (344 USD) per month for acquiring an identical better workplace. This character was particularly noticeable in the work environment incentive. In contrast to the 1107 CNY (170 USD) per month they were willing to denote at the basic-income level, at the higher-income level, they demonstrated a willingness to forgo an additional 992 CNY (153 USD) to achieve the identical enhanced work environment. Figure 2 illustrates the variation in the substitution effect of each non-financial incentive across various income levels.

### Heterogeneity analysis on altruism

The altruism *α* was quantified through the quantity choices made in the lab-like experiment and the utility function. The median value of *α* stood at 0.89. Further, we divided the participants into two groups based on the median of altruism. Distinguishing between levels of intrinsic altruism, we analyzed heterogeneity in job preferences for each incentive across income levels.

**Fig. 3:**
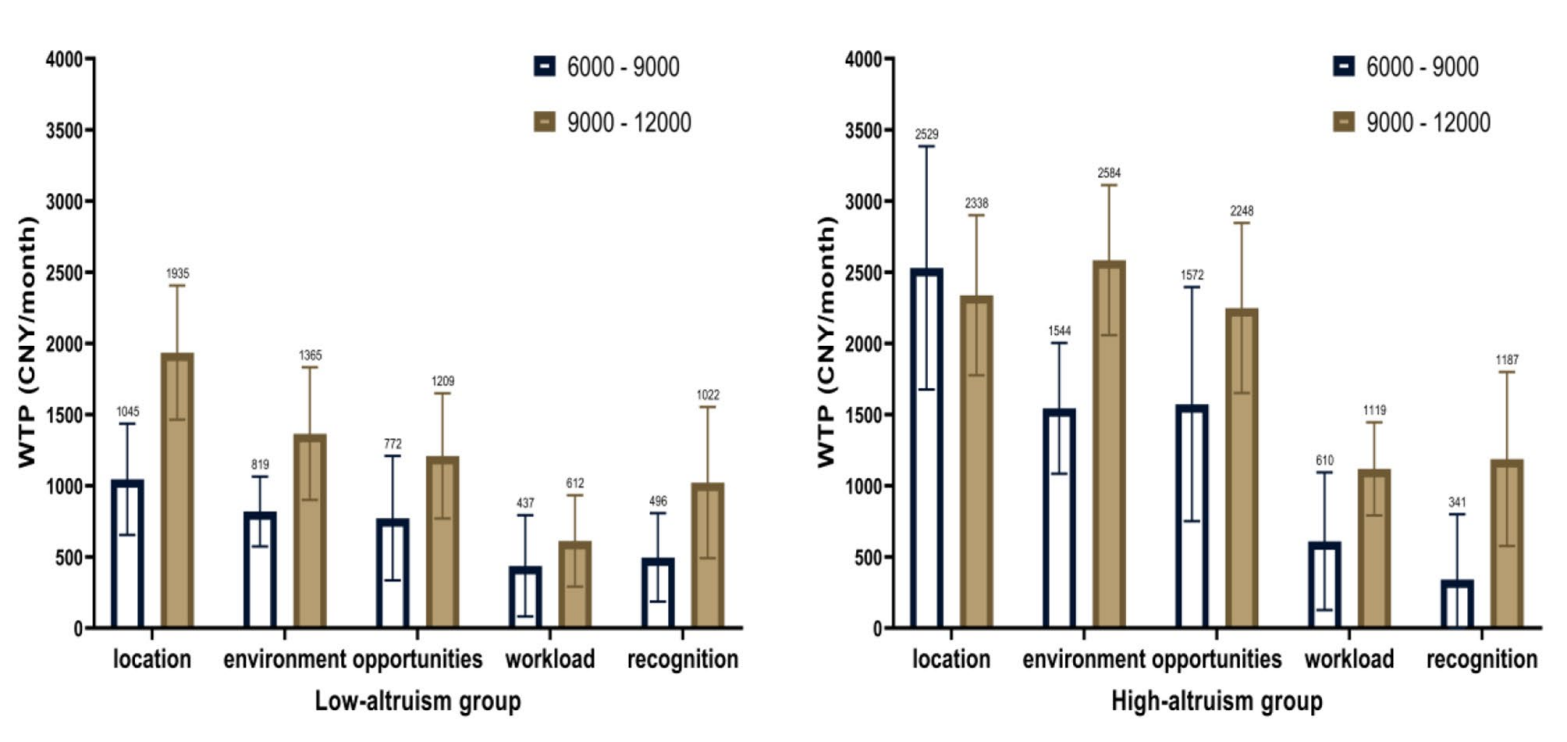
The substitution of non-financial and financial incentives of subgroups at different income levels with different levels of altruism. location: Work location; environment: Work environment; opportunities: Training and career development opportunities; workload: Workload; recognition: Professional recognition. The exchange rate for USD/CNY = 6.5

The results showed that the substitution effect in the high-altruism group was greater than that in the low-altruism at the same income level (see Fig. 2). Within the low-altruism group, the substitution effect demonstrated an upward trend across all attributes with increasing income levels. The workplace attribute exhibited the most substantial increase in WTP, amounted to 890 CNY (140 USD), observing a shift from 1045 CNY (160 USD) per month at the basic-income level to 1935 CNY (298 USD) per month at the higher-income level (see Fig. 3; Table 3).

**Table 3:** Estimation for job preferences in low-altruism group

	(1) 6000 vs. 9000 CNY				(2) 9000 vs. 12,000 CNY			
Incentive factors	Coeff.	SE	WTP	95% CI	Coeff.	SE	WTP	95% CI
Monthly income	0.000572***	5.26e-05			0.000410***	4.41e-05		
Work location: village or township (ref)	0.598***	0.106	1045	(654,1435)	0.794***	0.0768	1935	(1464,2406)
Work environment: poor (ref)	0.469***	0.0820	819	(574,1063)	0.561***	0.104	1365	(900,1831)
Training and career development opportunities: insufficient (ref)	0.442***	0.1000	772	(335,1209)	0.496***	0.0759	1209	(769,1650)
Workload: 60 h/week (ref)	0.251*	0.118	437	(81,793)	0.251**	0.0810	612	(291,933)
Professional recognition: low (ref)	0.284**	0.0973	496	(185,806)	0.420***	0.0804	1022	(491,1553)
N	329				329			
Observation	3921				3894			
Log likelihood	-1124.2131				-1056.7337			
Pseudo R^2^	0.2171				0.2590			
LR χ^2^	623.35				738.53			
Prob > χ^2^	< 0.0001				< 0.0001			

^***^P < 0.001, ^**^P < 0.01, ^*^P < 0.1. Coeff: mean estimated coefficient; SE: standard error. Since monthly income was treated as a continuous variable, its estimated coefficient was less than 0.001. The exchange rate for USD/CNY = 6.5.

However, disparities in trends surfaced within the high-altruism group. The substitution effect of work location did not exhibit a significant increase across varying income levels. WTP was 2529 CNY (390 USD) per month at basic-income level, while it decreased to 2338 CNY (360 USD) per month at the higher-income level. Except for the workplace, the substitution effects of the remaining non-financial incentives in the high-altruism group exhibited an upward trend with increasing income levels. The most notable change occurred in the work environment, escalating from 1544 CNY (238 USD) per month at the basic-income level to 2584 CNY (397 USD) per month at the higher-income level. Under equivalent proportions of income growth, the fluctuation in the substitution effect was greater in the high-altruism group than in the low-altruism. Regarding the work environment, as income increased from the basic to the higher level, the low-altruism group experienced a growth of 546 CNY (84 USD) per month in the substitution effect, whereas the high-altruism group exhibited a growth of 1040 CNY (160 USD) per month (see Fig. 3; Tables 3 and 4).

**Table 4:** Estimation for job preferences in high-altruism group

	(1) 6000 vs. 9000 CNY				(2) 9000 vs. 12,000 CNY			
Incentive factors	Coeff.	SE	WTP	95% CI	Coeff.	SE	WTP	95% CI
Monthly income	0.000362***	4.55e-05	2529	(1675,3384)	0.000386***	4.56e-05		
Work location: village or township (ref)	0.916***	0.110	1544	(1084,2003)	0.902***	0.0709	2338	(1776,2899)
Work environment: poor (ref)	0.559***	0.0784	1572	(750,2394)	0.997***	0.106	2584	(2057,3112)
Training and career development opportunities: insufficient (ref)	0.570***	0.0973	610	(126,1093)	0.867***	0.0768	2248	(1651,2846)
Workload: 60 h/week (ref)	0.221*	0.106	341	(-116,798)	0.432***	0.0850	1119	(792,1446)
Professional recognition: low (ref)	0.124	0.0887	2529	(1675,3384)	0.458***	0.0823	1187	(577,1798)
N	366				366			
Observation	4338				4380			
Log likelihood	-1264.8729				-1145.517			
Pseudo R^2^	0.2038				0.2858			
LR χ^2^	647.44				916.91			
Prob > χ^2^	< 0.0001				< 0.0001			

^***^P < 0.001, ^**^P < 0.01, ^*^P < 0.1. Coeff: mean estimated coefficient; SE: standard error. Since monthly income was treated as a continuous variable, its estimated coefficient was less than 0.001. The exchange rate for USD/CNY = 6.5.

## Discussion

Consistent with similar works we found a positive relationship between financial incentives and medical students’ job choices [30, 31]. However, with the upward trend in the monthly income, the results showed that jobs with same non-financial attributes consistently had higher utility, which demonstrated financial incentives were not as the most effective interventions underlying job preferences as previously believed. This outcome was also found with DCEs in Tanzania [32], Mozambique [21] and Malawi [26], suggesting the evidence of the diminishing marginal utility of income. The hierarchy of needs theory points that the lower needs must be externally satisfied through financial incentives [33]. When the income was relatively low, the marginal utility of unit monetary compensation was higher. In such circumstances, employees were more inclined to exert their maximum effort to acquire the increase in their monetary compensation. As income level increased, the lower needs were gradually satisfied and the higher-order needs became dominant. In our study, monthly income was set based on the actual figures, once remuneration reached a level allowing medical students to meet basic needs, the utility increase of financial incentives was smaller as income moved to the higher level, thus making other non-financial incentives become more compelling.

In particular, better work location, work environment and career development were the most important non-financial incentives. This outcome was consistent with the previous DCEs [34–36]. Despite the evidence in Blaauw et al. (2010) suggested that urban location may not be a high priority for health workers [37], there continues to be a strong emphasis on incentivizing them to rural location. Survey conducted in Indonesia [38], Uganda [39], and Nigeria [40] emphasized the importance of policy interventions that focused on providing a supportive management and an advanced facility as effective approaches. Our findings also suggested that the quality of a favorable work environment and sufficient career development were linked to the satisfaction of health workers, particularly when income reached a higher level. The work environment, as defined in this study, also included superior interpersonal relationships and organizational culture. Thus, more attention should be given to the internal cohesion and development prospects in medical institutions.

The self-determination theory (SDT) assert that individuals’ career attention is motivated by both internal and external conditions [41]. Similar results were found in this study that the both intrinsic altruism as well as external incentives contribute to job preference. Piatak (2015) indicated that a positive association existed between intrinsic altruism and the public sector job preference [42], while salary was a commonly important motivator for students who prefer either not-profit or for-profit jobs [43]. This study also found the role of altruism, and further identified medical students with higher altruism inclined to pay more attention to non-financial incentives, aligning with the results that health workers who prioritize intrinsic motivators over extrinsic ones were associated with a lower likelihood of turnover and greater job satisfaction [44]. At higher income level, the high-altruism group placed greater emphasis on the better working environment even over work location. Several studies indicated that a positive association between supportive work environment and intrinsic motivation [45]. The average altruistic parameter of 0.84 in our study exceeded 0.75 in Brosig-Koch et al. (2017) [28], further we demonstrated that altruism in medical students was significantly higher than non-medical students (0.60). The altruism among medical students was relatively higher, which may be one factor contributing to the importance of working environment. Earlier studies showed that health workers with higher altruism were more likely to accept work in rural locations [46–48]. Simultaneously, urban geographic location was at times associated with the highest salaries [49]. With the increase in income, these factors could potentially contribute even more to the diminished relative importance of work location within the high-altruism medical students.

In line with the hypothesis, individuals with higher altruism experienced elevated growth in the substitution effect of non-financial incentives on financial incentives as income increased. Consistent with the previous economic experiment [28], altruism in this study was also defined as medical students’ weight on patients’ benefit. Altruistic individuals themselves paid more attention to non-financial incentives, thereby increasing the degree of diminishing marginal utility of income.

## Conclusions

This study offers a comprehensive perspective that income plays a role in substitution of non-financial and financial incentives, currently also influenced by intrinsic altruism of medical students. The results of this study demonstrate that the non-financial incentives become relatively more important as the income level increases. Additionally, the substitution effect of non-financial incentives is more pronounced among medical students with higher altruism. Therefore, policymakers and hospital managers should prioritize ensuring a higher income monetary compensation to maintain the motivation of health workers. On basis of this, it is advisable to appropriately integrate non-financial incentives, such as offering a favorable working environment and promising career prospects, to effectively address the diminishing marginal effectiveness of monetary compensation among medical students. Furthermore, medical school administrations should focus on promoting altruistic values in medical education and enhancing health talent incentive strategies to encourage medical students to devote themselves to professions related to medicine.

### Limitations

This study has three limitations. Firstly, the data for this study are derived from teaching hospitals in Beijing. Although the medical students from Beijing will work in the nationwide, these teaching hospitals may not fully represent the national actual situation, and the international generalization is limited. Secondly, the results were derived from stated preference rather than revealed preferences, which might diverge when individuals were faced with real-life situations. Thirdly, the representation of monthly income in this study was confined to specific points, whereas actual income is continuous. Identifying critical points in income required further exploration through empirical study.

### Electronic supplementary material

Below is the link to the electronic supplementary material.

Supplementary Material 1

## Data Availability

The data for this study are available from the corresponding author, YLH, upon reasonable request.

## Footnotes

1 The average exchange rate for USD/CNY is 6.5.
