# Supplementary material for: The substitution effect of financial and non-financial incentives at different income levels in physician recruitment: evidence from medical students in China
Source: BMC Med Educ. 2024 May 9;24:503. doi: 10.1186/s12909-024-05374-6 (PMC11080174; doi:10.1186/s12909-024-05374-6)
Supplement: Supplementary file 1 — Supplementary Material 1 [file 12909_2024_5374_MOESM1_ESM.docx]

# Appendix

**Table S1** The formula for utility function and substitution effect in job preference

| The formula | Explanation |
| --- | --- |
| $U_{i}=\left( 1-\mu\right)U_{M}\left( i \right)+\mu U_{N}\left( i \right)$ | The utility function for job preference, consisting of both financial ($M$) and non-financial ($N$) incentives at different income levels ($i)$ |
| $U_{M}\left( i \right)={(i^{*}-i)}^{2}$ | The utility function ($U_{M}$) for financial incentives and income level, attaining its maximum at $i^{*}$ |
| $U_{N}\left( i \right)=pi$ | The utility function ($U_{N}$) for non-financial incentives and income level |
| ${MRS}_{MN}=\frac{2\left( i-i^{*} \right)}{p}$ | The substitution effect between non-financial and financial incentives |

**Table S2** The formula for utility function and altruism in medical decision-making

| The formula | Explanation |
| --- | --- |
| $U\left( q \right)=(1-\alpha)\pi\left( q \right)+\alpha B\left( q \right)$ | The utility functions for physicians’ medical decision-making, consisting of both physician profit $\pi\left( q \right)$ and patient benefit $B\left( q \right)$. Altruism is represented as $\alpha$. |

**Table S3** Attributes and levels for DCE

| Attributes | Definition | Levels |
| --- | --- | --- |
| Monthly income | Monthly income refers to pre-tax income, including basic salary, bonuses, and various benefits. | 6000 CNY/month |
|  |  | 9000 CNY/month |
|  |  | 12,000 CNY/month |
| Work location | Work location refers to work in various medical and health institutions in different regions. | Village or township |
|  |  | County |
|  |  | City |
| Work environment | Work environment includes access to needed equipment and supplies, such as medications, and support from management, amenities, and positive interpersonal relationships. | Poor |
|  |  | Common |
|  |  | Excellent |
| Training and career development opportunities | Training and career development opportunities refer to professional title assessment, career promotion, short-term training, and long-term continuing education. | Insufficient |
|  |  | General |
|  |  | Sufficient |
| Workload | Workload includes daily workload, overtime, on-duty hours, and night shifts. | 60 h/week |
|  |  | 50 h/week |
|  |  | 40 h/week |
| Professional recognition | Professional recognition refers to the degree of recognition of the valued placed on their jobs by the general public, patients, and families. | Low |
|  |  | Normal |
|  |  | High |

The exchange rate for USD/CNY = 6.5.

**Table S4** An example of one DCE choice set

| Attributes | Job A | Job B |
| --- | --- | --- |
| Monthly income | 12,000 CNY | 9000 CNY |
| Work location | Village or township | City |
| Work environment | Poor | Excellent |
| Training and career development opportunities | Insufficient | General |
| Workload | 50 h/week | 40 h/week |
| Professional recognition | High | Normal |
| Job preference |  |  |
| Job preference in real-life situation |  Yes | |
|  |  No | |

The exchange rate for USD/CNY = 6.5.

**Table S5** An example of one experimental task

| Medical service (Patient: *B_y_*) | Quantity | Payment | Cost | Profit | Patient benefit |
| --- | --- | --- | --- | --- | --- |
| None | 0 | 0 | 0 | 0 | 5 |
| Service 1 | 1 | 2 | 0.1 | 1.9 | 6 |
| Service 1, Service 2 | 2 | 4 | 0.4 | 3.6 | 7 |
| Service 1, …, Service 3 | 3 | 6 | 0.9 | 5.1 | 8 |
| Service 1, …, Service 4 | 4 | 8 | 1.6 | 6.4 | 9 |
| Service 1, …, Service 5 | 5 | 10 | 2.5 | 7.5 | 10 |
| Service 1, …, Service 6 | 6 | 12 | 3.6 | 8.4 | 9 |
| Service 1, …, Service 7 | 7 | 14 | 4.9 | 9.1 | 8 |
| Service 1, …, Service 8 | 8 | 16 | 6.4 | 9.6 | 7 |
| Service 1, …, Service 9 | 9 | 18 | 8.1 | 9.9 | 6 |
| Service 1, …, Service 10 | 10 | 20 | 10.0 | 10.0 | 5 |
| Please fill in the quantity of medical services you would like to provide (0–10): _____________ | | | | | |


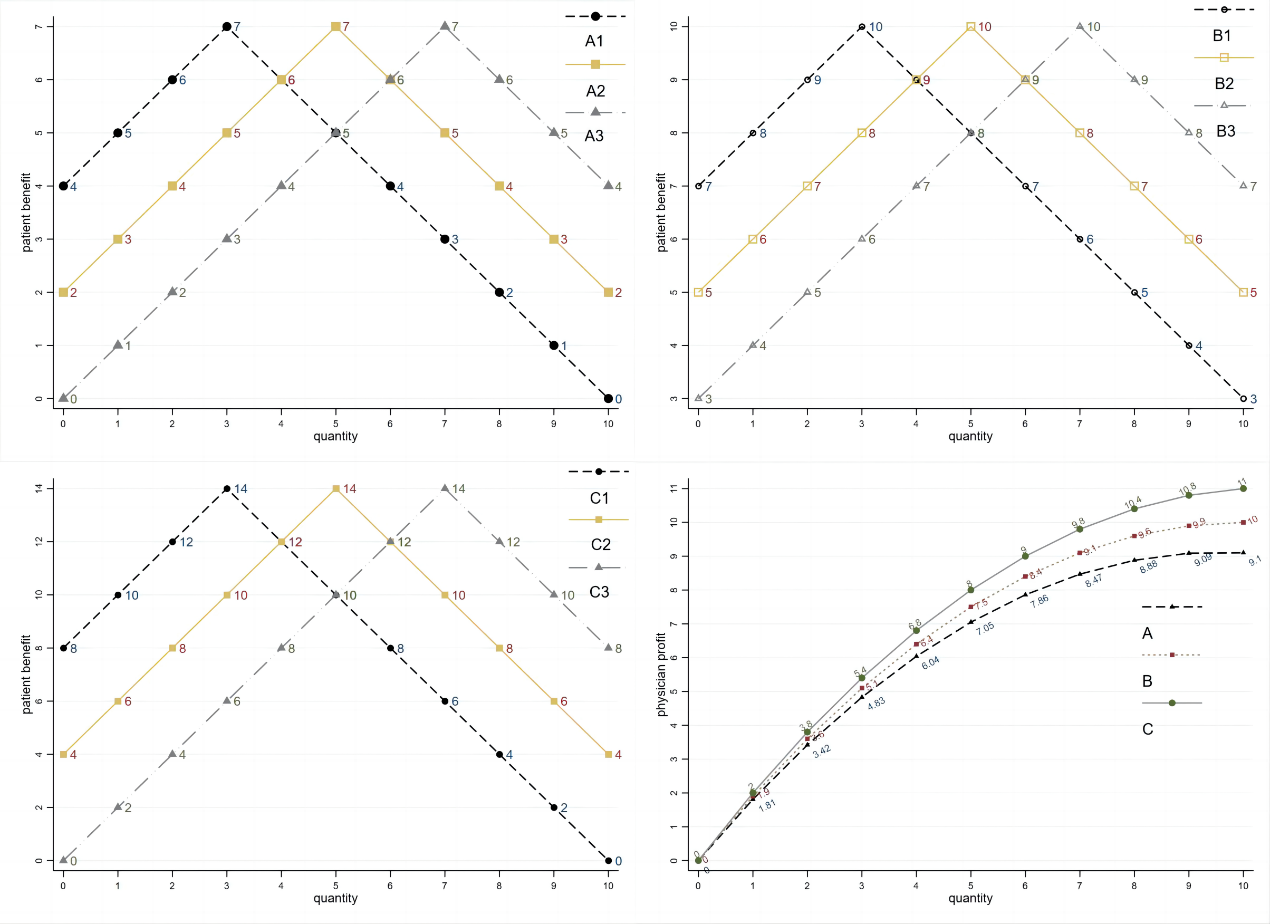


**Fig. S1** The experimental parameter of *B*(*q*) and *π*(*q*)

**Table S6** Standardized altruism *α* based on the quantity choices

| quantity | 0 | 1 | 2 | 3 | 4 | 5 | 6 | 7 | 8 | 9 | 10 |
| --- | --- | --- | --- | --- | --- | --- | --- | --- | --- | --- | --- |
| *A*_1_ | ‒ | ‒ | ‒ | 1.00 | 0.93 | 0.84 | 0.73 | 0.60 | 0.42 | 0.17 | 0.00 |
| *A*_2_ | ‒ | ‒ | ‒ | ‒ | ‒ | 1.00 | 0.87 | 0.71 | 0.50 | 0.21 | 0.00 |
| *A*_3_ | ‒ | ‒ | ‒ | ‒ | ‒ | ‒ | ‒ | 1.00 | 0.70 | 0.29 | 0.00 |
| *B*_1_ | ‒ | ‒ | ‒ | 1.00 | 0.94 | 0.86 | 0.76 | 0.64 | 0.49 | 0.29 | 0.00 |
| *B*_2_ | ‒ | ‒ | ‒ | ‒ | ‒ | 1.00 | 0.89 | 0.75 | 0.57 | 0.33 | 0.00 |
| *B*_3_ | ‒ | ‒ | ‒ | ‒ | ‒ | ‒ | ‒ | 1.00 | 0.76 | 0.44 | 0.00 |
| *C*_1_ | ‒ | ‒ | ‒ | 1.00 | 0.92 | 0.83 | 0.72 | 0.60 | 0.47 | 0.30 | 0.00 |
| *C*_2_ | ‒ | ‒ | ‒ | ‒ | ‒ | 1.00 | 0.87 | 0.73 | 0.56 | 0.37 | 0.00 |
| *C*_3_ | ‒ | ‒ | ‒ | ‒ | ‒ | ‒ | ‒ | 1.00 | 0.77 | 0.50 | 0.00 |

**Table S7** Estimation of mixed logit model for employment preference

|  | (1) Analysis Sample | | (2) 6000 *vs* 9000 CNY | | (3) 9000 *vs*12000 CNY | |
| --- | --- | --- | --- | --- | --- | --- |
| Incentive factors | Coeff. (SE) | SD (SE) | Coeff. (SE) | SD (SE) | Coeff. (SE) | SD (SE) |
| Monthly income | 0.000549*** | 0.000111*** | 0.000484*** | -0.000131** | 0.000552*** | -5.67e-05 |
|  | (1.52e-05) | (1.26e-05) | (4.28e-05) | (3.98e-05) | (4.28e-05) | (3.93e-05) |
| Work location:  village or township (ref) | 1.244*** | 0.836*** | 1.103*** | -0.290 | 1.129*** | -0.466*** |
|  | (0.0492) | (0.0492) | (0.109) | (0.207) | (0.0739) | (0.0865) |
| Work environment: poor (ref) | 0.827*** | 0.0813 | 0.574*** | -0.181 | 1.109*** | -0.315+ |
|  | (0.0377) | (0.0907) | (0.0784) | (0.155) | (0.0965) | (0.166) |
| Training and career development opportunities: insufficient (ref) | 0.697*** | 0.722*** | 0.748*** | 0.776*** | 0.892*** | -0.512*** |
|  | (0.0434) | (0.0481) | (0.106) | (0.116) | (0.0749) | (0.0827) |
| Workload: 60 h/week (ref) | 0.473*** | 0.384*** | 0.126 | -0.746*** | 0.595*** | -0.223+ |
|  | (0.0330) | (0.0493) | (0.111) | (0.133) | (0.0759) | (0.114) |
| Professional recognition: low (ref) | 0.494*** | 0.416*** | -0.00641 | -0.388** | 0.417*** | 0.0248 |
|  | (0.0361) | (0.0569) | (0.0894) | (0.126) | (0.0678) | (0.105) |
| N | 741 | | 741 | | 741 | |
| Observation | 26,559 | | 8,796 | | 8,850 | |
| Log likelihood | -6036.5841 | | -2234.2236 | | -2226.5475 | |
| LR χ^2^ | 2594.32 | | 679.25 | | 294.72 | |
| Prob > χ^2^ | < 0.0001 | | < 0.0001 | | < 0.0001 | |
| AIC | 12101.17 | | 4496.447 | | 4481.095 | |
| BIC | 12215.79 | | 4595.596 | | 4580.329 | |

^***^*P* < 0.001, ^**^ *P* < 0.01, ^*^ *P* < 0.1. Coeff: mean estimated coefficient; SE: standard error; SD: standard deviation, indicating preference heterogeneity; AIC: Akaike Information Criterion; BIC: Bayesian Information Criterion. Since monthly income was treated as a continuous variable, its estimated coefficient was less than 0.001. The exchange rate for USD/CNY = 6.5.
